# *Colletotrichum* Species Causing Anthracnose of Citrus in Australia

**DOI:** 10.3390/jof7010047

**Published:** 2021-01-12

**Authors:** Weixia Wang, Dilani D. de Silva, Azin Moslemi, Jacqueline Edwards, Peter K. Ades, Pedro W. Crous, Paul W. J. Taylor

**Affiliations:** 1 Faculty of Veterinary and Agricultural Sciences, The University of Melbourne, Parkville, VIC 3010, Australia; weixiaw@student.unimelb.edu.au (W.W.); dilani.desilva@ecodev.vic.gov.au (D.D.d.S.); Azin.moslemi@curtin.edu.au (A.M.); 2 Agriculture Victoria, Department of Jobs, Precincts and Regions, AgriBio Centre, 5 Ring Road, La Trobe University, Bundoora, VIC 3083, Australia; Jacky.Edwards@agriculture.vic.gov.au; 3 School of Applied Systems Biology, La Trobe University, Bundoora, VIC 3083, Australia; 4 Faculty of Science, The University of Melbourne, Parkville, VIC 3010, Australia; petera@unimelb.edu.au; 5 Westerdijk Fungal Biodiversity Institute, Uppsalalaan 8, 3584 CT Utrecht, The Netherlands; p.crous@wi.knaw.nl

**Keywords:** anthracnose, citrus, *Colletotrichum australianum*, phylogenetic analysis, taxonomy

## Abstract

*Colletotrichum* spp. are important pathogens of citrus that cause dieback of branches and postharvest disease. Globally, several species of *Colletotrichum* have been identified as causing anthracnose of citrus. One hundred and sixty-eight *Colletotrichum* isolates were collected from anthracnose symptoms on citrus stems, leaves, and fruit from Victoria, New South Wales, and Queensland, and from State herbaria in Australia. *Colletotrichum australianum* sp. nov., *C. fructicola*, *C. gloeosporioides*, *C. karstii, C. siamense*, and *C. theobromicola* were identified using multi-gene phylogenetic analyses based on seven genomic loci (ITS, *gapdh*, *act*, *tub2*, *ApMat*, *gs*, and *chs-1*) in the gloeosporioides complex and five genomic loci (ITS, *tub2*, *act*, *chs-1*, and *his3*) in the boninense complex, as well as morphological characters. Several isolates pathogenic to chili (*Capsicum annuum*), previously identified as *C. queenslandicum*, formed a clade with the citrus isolates described here as *C. australianum* sp. nov. The spore shape and culture characteristics of the chili and citrus isolates of *C. australianum* were similar and differed from those of *C. queenslandicum*. This is the first report of *C. theobromicola* isolated from citrus and the first detection of *C. karstii* and *C. siamense* associated with citrus anthracnose in Australia.

## 1. Introduction

Edible citrus (*Citrus* spp.) are important fruit crops globally, produced in temperate and tropical climates [1]. Cumquat (*Citrus japonica*), grapefruit (*Citrus × paradisi*), lemon (*Citrus limon*), lime (*Citrus aurantifolia*), mandarin (*Citrus reticulata)*, and orange (*Citrus × sinensis*) are all commercially important citrus species [1,2]. Australia is a major citrus producer with citrus grown in every mainland state [3,4]. In 2019, there was approximately 25,500 ha of citrus production in Australia [5]. Citrus is one of the largest fresh fruit exports from Australia. Australia exported 251,594 tonnes of citrus in 2018, with a total value of $A452.9 million [6].

In citrus, anthracnose caused by *Colletotrichum* spp. is a serious disease limiting production globally. Preharvest anthracnose reduces yield, while postharvest anthracnose affects fruit quality, negatively impacting fruit export and marketability [7]. *Colletotrichum* species are difficult to identify based on morphological characters. Molecular phylogeny has reinvigorated *Colletotrichum* taxonomy [8], with over 220 *Colletotrichum* species in 14 species complexes now recognised [9,10].

Globally, multiple *Colletotrichum* species within several species complexes have been identified as causing citrus anthracnose. *Colletotrichum gloeosporioides* was reported to be associated with anthracnose in Australia [8], Vietnam [11], China [12], Italy [8,13], Morocco [14], Mexico [15,16], Pakistan [17], Ghana [18,19], Brazil [11,20], Algeria [21], Greece [8], Malta [8], New Zealand [8], Portugal [8,22], South Africa [11], Spain [8], Tunisia [23,24], United States [8] and Zimbabwe [11]. *Colletotrichum karstii* was reported in Southern Italy [13], China [25,26,27], Portugal [23], South Africa [11], Europe [8], United States [28], Tunisia [16], Turkey [29], and New Zealand [25]; *C. fructicola* was reported in China [26,27,30]; and *C. siamense* was reported in Vietnam [11], Bangladesh [11], Egypt [11], China [31], Mexico [22], and Pakistan [17,32]. Additionally, *C. abscissum, C. acutatum, C. boninense, C. brevisporum, C. catinaense, C. citri, C. citricola*, *C. citri-maximae, C. constrictum, C. godetiae, C. helleniense*, *C. hystricis, C. johnstonii, C. cigarro, C. limetticola*, *C. limonicola, C. novae-zelandiae, C. queenslandicum, C. simmondsii, C. tropicicola*, and *C. truncatum* have all been associated with citrus anthracnose [8,11,25,27,33,34,35,36].

*Colletotrichum acutatum*, *C. fructicola*, *C. gloeosporioides*, and *C. nymphaeae* have been reported as pathogens associated with citrus anthracnose in Australia. However, *C. acutatum* was identified based on morphology, and *C. nymphaeae* was verified by a single *tub2* sequence [37,38]. Citrus fruits and plants with anthracnose symptoms are very common both in home gardens and in commercial orchards in Australia. Hence, it is necessary to accurately characterize the *Colletotrichum* species causing anthracnose diseases of citrus in Australia to help develop appropriate disease management strategies and provide a baseline for plant biosecurity, trade, and market access.

In this study, a representative collection of *Colletotrichum* isolates from eastern Australian citrus was established from symptomatic leaves, twigs, and fruit, and from culture collections. *Colletotrichum* species were determined by utilising a polyphasic approach, in which informative gene loci were sequenced. Multigene phylogenetic analyses, morphological characters, and pathogenicity bioassays were used to confirm the taxonomy and phylogenetic relationships of *Colletotrichum* spp. pathogens causing citrus anthracnose in Australia.

## 2. Materials and Methods

### 2.1. Sample Collection

A total of 147 *Colletotrichum* isolates were collected from anthracnose lesions on citrus stems and leaves of trees growing in Victoria and New South Wales and from citrus fruits with anthracnose disease symptoms from supermarkets in Melbourne, Victoria. In addition, 21 isolates originating from citrus plants were obtained from State fungaria (the Victorian Plant Pathology Herbarium (VPRI), the Queensland Plant Pathology Herbarium (BRIP), and the NSW Plant Pathology Collection (DAR)).

### 2.2. Isolate Preparation

Infected fruits, stems, and leaves were surface sterilized by dipping in 2.3% (active ingredient) sodium hypochlorite (NaOCl) for 2 min and rinsed five times with sterile distilled water (SDW). Tissue pieces (2 mm^2^) were excised from the margins of infected lesions and plated onto potato dextrose agar (PDA). The plates were incubated at 25 °C in continuous dark for 7 d as described by Guarnaccia et al. [8]. Subcultures of mycelia on PDA plates were maintained under the same growing conditions for a further 7 d. All isolates were established as single spore cultures, as described in De Silva et al. [39].

### 2.3. Morphological and Cultural Analyses

Plugs (2 mm^2^) of actively growing mycelia were taken from the edge of 7-d-old cultures and transferred onto PDA and synthetic nutrient-poor agar (SNA), as described by Guarnaccia et al. [8]. After 7 d of incubation at 25 °C under continuous near-ultraviolet light, colony growth was determined by measuring two diameters perpendicular to each other per plate and determining the average of six plates. At 10 d, colony colour was determined using colour charts [40]. Acervuli were induced by inoculating pieces of sterilized mandarin rind with mycelia and incubating on water agar (WA) and SNA, at 25 °C for 10 d.

Appressoria were induced using the slide culture technique described by Johnston and Jones [41]. The length and width of 30 appressoria/slide were measured using X1000 magnification with a Leica DM6000 LED compound microscope, Leica DMC2900 camera, and Leica LAS v. 4.5.0 software.

Slide preparations of morphological structures were prepared in lactic acid, and at least 30 observations were recorded for conidia, conidiophores, and conidiogenous cells per isolate, as well as presence or absence of setae. The range, mean, and standard error (SE) were calculated for each isolate.

### 2.4. Multigene Phylogenetic Analysis

#### 2.4.1. DNA Extraction, PCR Amplification, and Sequencing

1. DNA extraction

Genomic DNA was extracted from pure (single-spored) mycelia of *Colletotrichum* isolates grown on PDA at 25 °C for 7 d using DNeasy Plant Mini kits (Qiagen, Australia), following the manufacturer’s instructions. DNA concentration was determined using NanoDrop, then diluted to 2 ng∙µL^−1^ and stored at −20 °C until further use [39].

2. PCR amplification and sequencing

Isolates were assigned to a species complex based on morphology and internal transcribed spacer and intervening 5.8S nrDNA gene (ITS) and β-tubulin (*tub2*) gene sequences data. Isolates in the gloeosporioides species complex were further characterised using seven gene loci: ITS, glyceraldehyde-3-phosphate dehydrogenase (*gapdh*), actin (*act*), *tub2*, the Apn2–Mat1–2 intergenic spacer and partial mating type (Mat1–2) (*ApMat*), glutamine synthetase (*gs*), and chitin synthase 1 (*chs-1*) genes. Isolates in the boninense species complex were further characterised using five gene loci: ITS, *tub2*, *act*, *chs-1*, and histone (*his3*). These gene sequences were amplified and sequenced by using primer pairs: ITS-1F (ITS; [42]) and ITS4 (ITS; [43]), GDF1 and GDR1 (*gapdh*; [44]), ACT-512F + ACT-783R (*act*; [45]), Btub2Fd and Btub4Rd (*tub2*; [46]), AMF1 and AMR1 (*ApMat*; [47]), GSF1 and GSR1 (*gs*; [48]), CHS-79F and CHS-354R (*chs-1*; [45]), and CYLH3F and CYLH3R (*his3*; [49]).

PCR was performed in a 2720 Thermal Cycler (Applied Biosystems, Australia). The total volume of PCR mixture was 25 µL. The PCR of the ITS, *gapdh*, *act*, *tub2*, *gs*, *chs-1,* and *his3* genes followed the protocol described by De Silva et al. [39] and contained 1× PCR buffer, 2 mM MgCl_2_, 0.2 mM dNTP, 1 U *Taq* DNA polymerase (Mango*Taq* DNA polymerase; Bioline, Australia), 0.4 µM of each primer, and 6 ng template DNA. The PCR annealing temperatures were adjusted to 55 °C for ITS, *gapdh,* and *his3*; 58 °C for *act*, *tub2,* and *gs*; and 66 °C for *chs-1*.

For *ApMat*, in the 25 µL PCR mixture, the concentration of each primer was adjusted to 0.5 µM, and the template DNA was adjusted to 10 ng. The PCR amplification protocols were performed according to Silva et al. [47], except the annealing temperature of *ApMat* was adjusted to 62 °C.

All PCR products were purified using QIA-quick PCR Purification Kit (Qiagen, Australia) following the manufacturer’s instructions. Purified PCR products were sequenced in both the forward and reverse sense at the Australian Genome Research Facility (AGRF, Melbourne), then aligned to produce a consensus sequence for each isolate using ClustalW in MEGA 6.06 [50]. The consensus sequences were deposited in GenBank.

#### 2.4.2. Phylogenetic Analyses

The sequences of reference isolates were retrieved from GenBank for use in phylogenetic analyses (Table 1). All the sequences were aligned by using ClustalW in MEGA 6.06 and manually edited when necessary. The ITS and *tub2* sequences of morphologically different isolates were compared to determine which species complex each isolate belonged based on maximum likelihood analysis (ML) by using MEGA 6.06 [10]. For isolates from the gloeosporioides species complex, phylogenetic analyses of combined seven gene sequences (ITS, *gapdh*, *act*, *tub2*, *ApMat*, *gs*, and *chs-1*) and combined two gene sequences (*ApMat* and *gs*) were carried out with selected reference sequences [39,51]. For isolates from the boninense species complex, phylogenetic analysis of combined five gene sequences (ITS, *tub2*, *act*, *chs-1*, and *his3*) was constructed [8].

Further phylogenetic analyses were based on Bayesian Inference analyses (BI) by using MrBayes v. 3.1.2 and ML analysis by using MEGA 6.06 [39]. For BI analyses, MrModeltest2.3 was used to determine the best-fit model for each locus [52] (Table 2). MrBayes v. 3.2.6 was used to generate phylogenetic trees. Four chains were used in the Markov Chain Monte Carlo (MCMC) analysis and were run for 1,000,000,000 generations. The trees were sampled every 100 generations and the heating parameter was set to 0.2. Analyses stopped once the average standard deviation of split frequencies was below 0.01. For ML analysis, analyses were done by using MEGA 6.06. The phylogeny test was the Bootstrap method with 1000 replicates. The substitution model was the Tamura–Nei model based on nucleotide type. The tree inference option was Nearest-Neighbor-Interchange (NNI) ML heuristic method.

### 2.5. Pathogenicity Testing

One isolate of each *Colletotrichum* species (except for *C. siamense*, which did not sporulate in culture) was used in the pathogenicity tests to inoculate orange (Washington Navel) fruits, orange leaves, lemon (Myer) leaves, and orange flower petals according to the method of Guarnaccia et al. [8].

#### 2.5.1. Fruit Bioassay

Conidial suspensions of each isolate were prepared by adding 10 mL of SDW to 7-d-old cultures, scraping the mycelia then filtering through muslin cloth. The concentration of spore suspension was adjusted to 10^6^ conidia mL^−1^. Organically grown orange fruits (*Citrus sinensis*) purchased from a market (Queen Victoria Market in Melbourne) were washed with tap water and then submerged in 70% ethanol for 10 min, and finally rinsed in SDW twice. The orange fruits were marked in the middle to divide into two parts and inoculated with both wound (W) and non-wound (NW) methods. For the wound method, the orange skin was pricked with a sterilized pipette tip to about 1 mm depth. Six wound points were made, and each inoculated with 6 µL spore suspension. In the non-wound method, six drops of 6 µL spore suspension were placed directly on the orange skin. For the control group, 6 µL of SDW was used to treat orange fruit in both wound and non-wound methods. There were three replicates per treatment per isolate and the experiments replicated twice. The fruit was transferred to a plastic box and incubated at 25 °C with 100% humidity in dark. After 10 d, fruits were examined for symptom development, and the percentage of infection was calculated ($\mathrm{percentage}\;\left(\%\right)=\frac{infected\;points}{inoculated\;points}\times 100\%$).

#### 2.5.2. Leaf Bioassay

Young, healthy, fully expanded orange and lemon leaves were collected from trees growing in pots. The leaves were washed with tap water, then submerged in 70% ethanol for 2 min, and finally rinsed in SDW twice. The petioles of leaves were wrapped with damp cotton wool and the leaves were placed into petri dishes, three leaves per dish. Three drops of 6 µL spore suspension (10^6^ conidia/mL) were individually placed directly onto the leaf upper surfaces. For the control group, 6 µL of SDW was used. Each set of three leaves per petri dish was inoculated with a different isolate. The petri dishes were placed inside a plastic box and the leaves incubated at 25 °C with 100% humidity and 12/12 h fluorescent light/dark cycle. After 10 d, the leaves were examined for symptom development, and the percentage of infection was calculated ($\mathrm{percentage}\;\left(\%\right)=\frac{infected\;points}{inoculated\;points}\times 100\%$).

#### 2.5.3. Petal Bioassay 

Healthy orange flower petals were collected from the same trees. Petals were washed in tap water, then submerged in 70% ethanol for 30 s, and finally rinsed in SDW twice. One drop of 6 µL spore suspension (10^3^ conidia/mL) was carefully placed on the middle of each petal without wounding. For the control group, 6 µL of SDW was used. Seven flower petals were used per isolate. The inoculated petals were put in a plastic box and incubated at 25 °C with 100% humidity and 12/12 h fluorescent light/dark cycle. After 3 d, the petals were examined for symptom development, and the percentage of infection was calculated ($\mathrm{percentage}\;\left(\%\right)=\frac{infected\;points}{inoculated\;points}\times 100\%$).

## 3. Results

### 3.1. Phylogenetic Analyses

The 147 isolates were separated into 18 morphological groups based on culture characteristics. One isolate from each morphological group and 18 isolates from State fungaria from different hosts and location were selected for phylogenetic analyses. Among the 36 *Colletotrichum* isolates, 29 were identified to be in the gloeosporioides complex and seven were identified to be in the boninense complex based on analysis of combined ITS and *tub2* gene sequences. All the isolates in the gloeosporioides complex were isolated from stems, leaves, or fruit, while six of the seven isolates in the boninense complex were isolated from infected orange leaf, while another one was from infected lemon leaf (Table S1).

#### 3.1.1. Gloeosporioides Species Complex

1. Seven-gene tree of citrus isolates in gloeosporioides species complex

**Figure 1:**
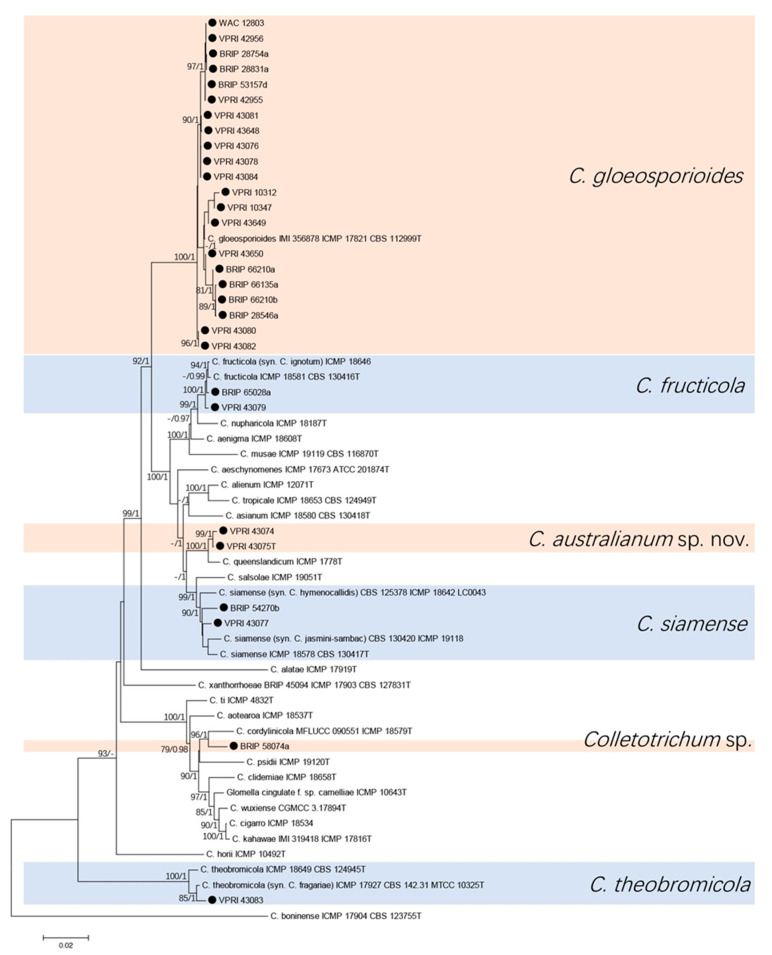
Phylogenetic analysis of the combined ITS, *gapdh*, *act*, *tub2*, *ApMat*, *GS*, and *chs-1* sequence alignment of *Colletotrichum* isolates in the gloeosporioides complex. The bootstrap support values (ML > 75%) of maximum likelihood analysis and Bayesian posterior probabilities (PP > 0.90) are displayed at the nodes (ML/PP). Black circle denotes isolates from *Citrus* spp.

The seven-gene phylogenetic analysis consisted of 29 citrus isolates and 29 reference sequences from the gloeosporioides species complex. *Colletotrichum boninense* (ICMP 17904T) was used as the out-group. A total of 3703 characters (ITS: 504, *gapdh*: 271, *act*: 271, *tub2*: 510, *ApMat*: 898, *gs*: 914, *chs-1*: 275 and 10 N to separate each two sequences) were analysed. The Bayesian analysis lasted 825,000 generations, resulting in 11,995 total trees, of which 8997 trees were used to calculate the posterior probabilities. The BI posterior probabilities were plotted on the ML tree (Figure 1).

2. Two-gene tree of citrus isolates in gloeosporioides species complex

**Figure 2:**
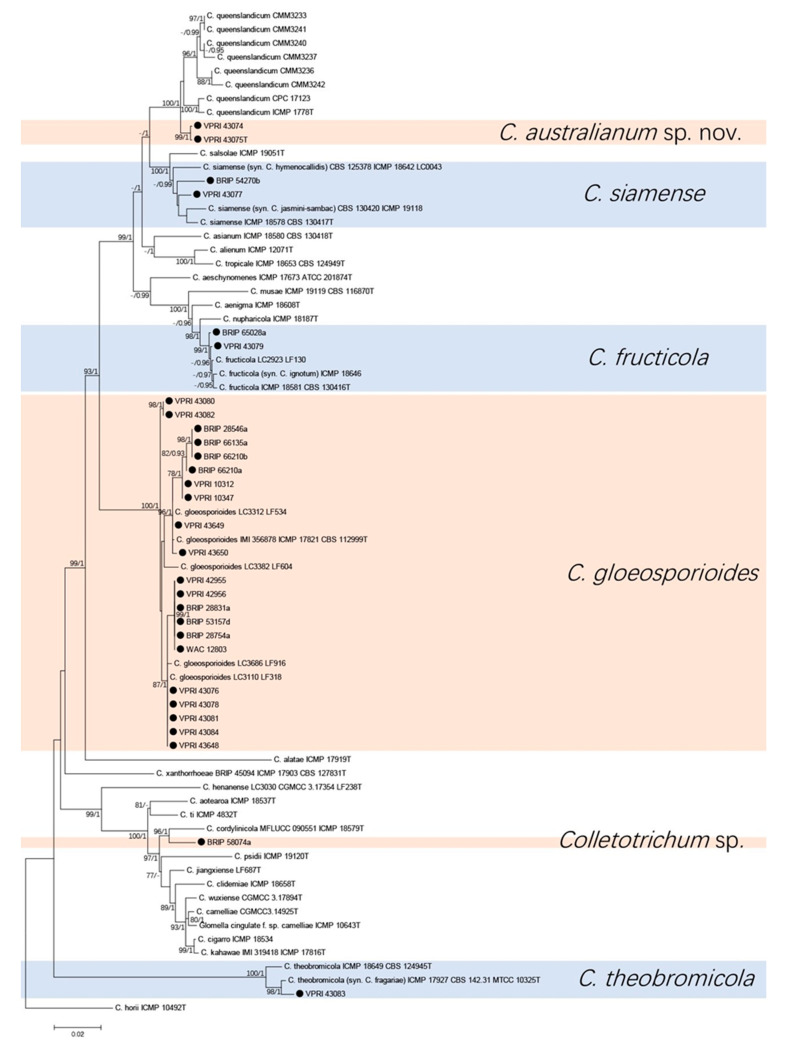
Phylogenetic analysis of the combined *ApMat* and *GS* sequence alignment of *Colletotrichum* isolates in the gloeosporioides complex. The bootstrap support values (ML > 75%) of maximum likelihood analysis and Bayesian posterior probabilities (PP > 0.90) are displayed at the nodes (ML/PP). Black circle denotes isolates from *Citrus* spp.

Analysis using the *ApMat* and *gs* sequence alignment consisted of 29 citrus isolates and 44 reference sequences from the gloeosporioides species complex. *Colletotrichum horii* (ICMP 10492T) was used as the out-group. A total of 1832 characters (*ApMat*: 903, *gs*: 919 and 10 N to separate two sequences) were analysed. The Bayesian analysis lasted 240,000 generations, resulting in 3601 total trees of which 2701 trees were used to calculate the posterior probabilities. The BI posterior probabilities were plotted on the ML tree (Figure 2).

Five species and one unknown *Colletotrichum* sp. were identified from the two trees (Figure 1 and Figure 2). Twenty-one (72%) of citrus isolates were identified as *C. gloeosporioides*, two isolates clustered with three reference isolates of *C. siamense*, two isolates clustered with three reference isolates of *C. fructicola*, and one isolate was identified to be *C. theobromicola*. Two isolates were identified and described as a new species, which was phylogenetically close but significantly different to *C. queenslandicum* with high support (100/1 in both trees). Isolate BRIP 58074a formed a significantly separate clade (96/1 in both trees) close to *C. cordylinicola*.

#### 3.1.2. Boninense Species Complex

**Figure 3:**
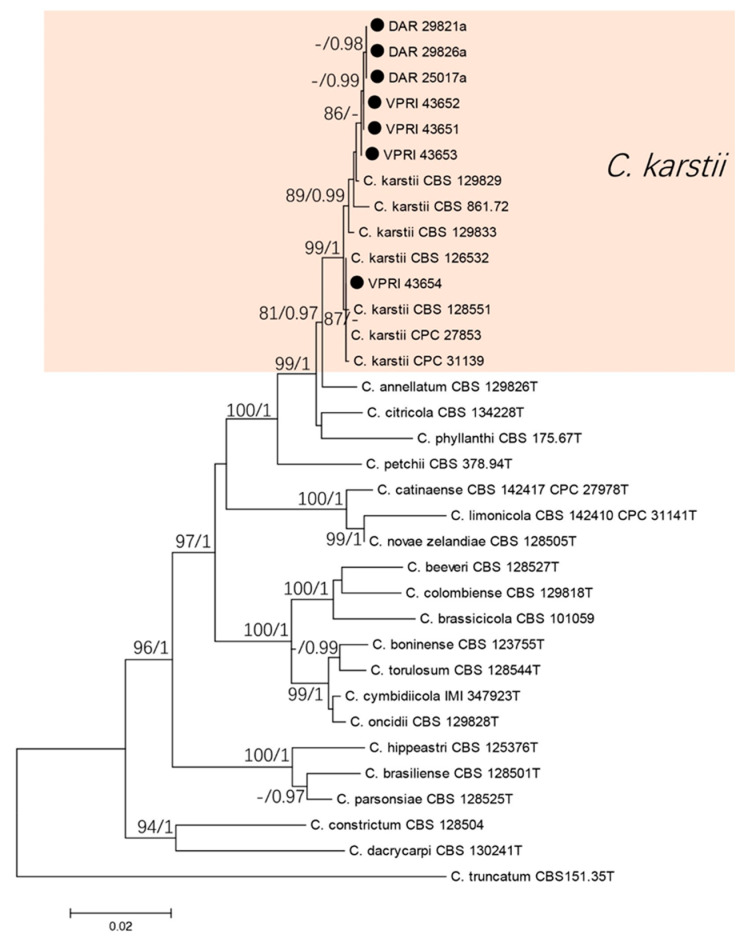
Phylogenetic analysis of the combined ITS, *tub2*, *act*, *chs-1*, and *his3* sequence alignment of *Colletotrichum* isolates in the boninense complex. The bootstrap support values (ML > 75%) of maximum likelihood analysis and Bayesian posterior probabilities (PP > 0.90) are displayed at the nodes (ML/PP). Black circle denotes isolates from *Citrus* spp.

The five gene phylogenetic analysis consisted of seven citrus isolates and 26 reference sequences from the boninense complex. *Colletotrichum truncatum* (CBS 151.35T) was used as the out-group. A total of 2048 characters (ITS: 559, *tub2*: 503, *act*: 280, *chs-1*: 282, *his3*: 395) were analysed. The Bayesian analysis lasted 135,000 generations, resulting in 1994 total trees, of which 1496 trees were used to calculate the posterior probabilities. The BI posterior probabilities were plotted on the ML tree. The phylogenetic analysis of the boninense species complex identified the seven citrus isolates as *C. karstii* (Figure 3).

### 3.2. Morphological Analysis

Morphological characters including conidial size, conidial shape, and growth rate of seven *Colletotrichum* species were recorded (Table 3). Their conidial size, conidial shape, and growth rate overlapped.

Morphological characters of the type specimen of *C. queenslandicum* (ICMP 1778) were according to Weir et al. [36] (Table 3). The new species varied morphologically from the type specimen of *C. queenslandicum* (ICMP 1778) by having different spore shape. Although the range of spore size overlapped between the new species and *C. queenslandicum*, the average conidial length of the new species was smaller than that of *C. queenslandicum* [36].

### 3.3. New Colletotrichum Species

#### 3.3.1. Two-Gene Tree of New Colletotrichum Species

**Figure 4:**
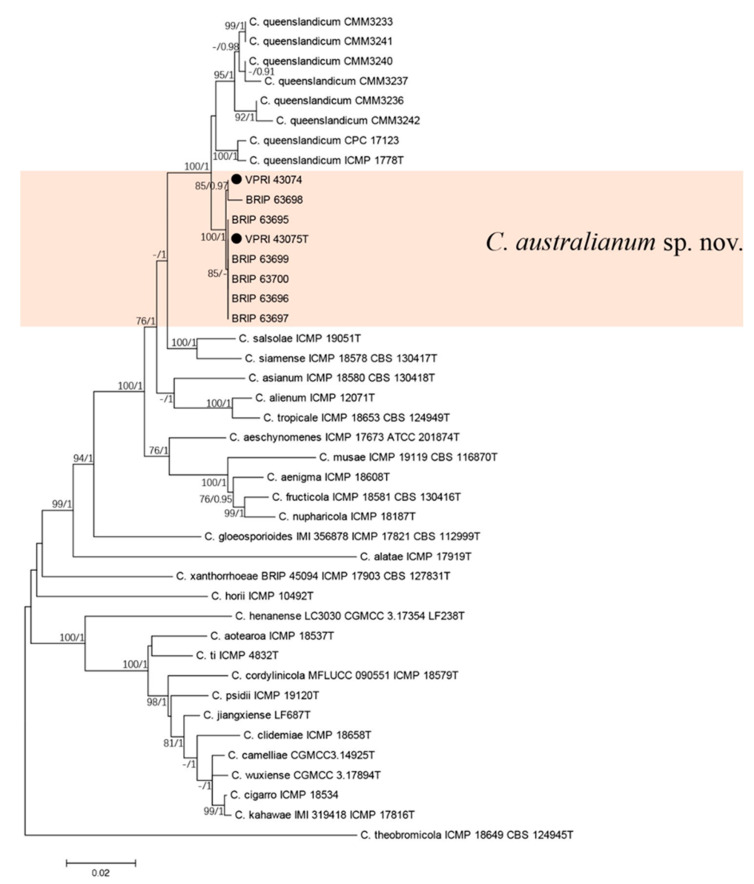
Phylogenetic analysis of the combined *ApMat* and *GS* sequence alignment of *Colletotrichum australianum* sp. nov. The bootstrap support values (ML > 75%) of maximum likelihood analysis and Bayesian posterior probabilities (PP > 0.90) are displayed at the nodes (ML/PP). Black circle denotes isolates from *Citrus* spp.

The two gene phylogenetic analysis consisted of six chili (*Capsicum annuum*) and two citrus isolates of the new *Colletotrichum* species, 34 reference sequences from the *C. gloeosporioides* species complex, including eight isolates of *C. queenslandicum*. *Colletotrichum theobromicola* (ICMP 18649T) was used as the out-group. A total of 1820 characters (*ApMat*: 900, *gs*: 910 and 10 N to separate two sequences) were analysed. The Bayesian analysis lasted 115,000 generations, resulting in 1709 total trees, of which 1282 trees were used to calculate the posterior probabilities. The BI posterior probabilities were plotted on the ML tree (Figure 4).

The six isolates from chili [39] clustered with the two citrus isolates of the new *Colletotrichum* species in the two-gene tree, which were significantly different from *C. queenslandicum* (Figure 4).

##### Taxonomy

Morphological characters and phylogenetic analyses indicated that the *Colletotrichum* species isolated from infected mandarin and orange fruits collected from Melbourne and Dunkeld, Victoria, respectively, and isolated from infected chili fruit collected from Brisbane, Queensland, Australia, was a new species, for which the name *Colletotrichum australianum* is proposed.

**Figure 5:**
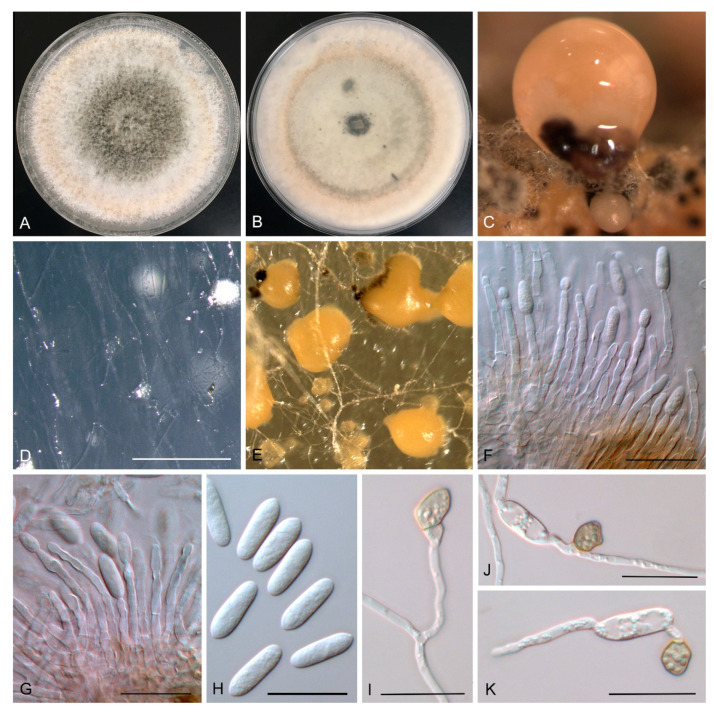
Morphological characteristics of *Colletotrichum australianum* sp. nov.: One-week-old culture on PDA (**A**,**B**), conidiomata on mandarin rind (**C**), conidiomata on SNA (**D**), conidiomata on PDA (**E**), conidiophores (**F**,**G**), conidia (**H**) and appressoria (**I**–**K**). Scale bars: D, 500 µm; F, G, H, I, J, K, 20 µm.

***Colletotrichum australianum*** W. Wang, D. D. De Silva, and P. W. J. Taylor, **sp. nov.** (Figure 5).

MycoBank Number: MB830323.

**Etymology:** Named after the country where the pathogen was first isolated, Australia.

**Holotype:** Australia, Victoria, Dunkeld, on fruit of *Citrus sinensis,* May 2016, *J. Kennedy* (VPRI 43075–holotype; UMC002–ex-type culture).

Asexual morph on SNA. Conidiomata on SNA inconspicuous or absent, 41–140 µm diam, formed from hyphae, lacking setae. Conidia hyaline, smooth, aseptate, straight, cylindrical with one end slightly acute, granular, and guttulate, (13.2–) 14.4–14.6 (–15.9) × (4.8–) 5.6–5.7 (–6.1) µm. Appressoria single, medium to dark brown, ovoid with an undulate margin, (6.1–) 8.5–8.9 (–12.2) × (4.6–) 6.7–7.1 (–9.3) µm.

Asexual morph on PDA. Conidiomata on PDA formed on hyphae or on a brown central stroma, lacking setae. Conidiophores hyaline, smooth-walled, septate, branched, 28–58 × 2–3 μm. Conidiogenous cells hyaline, smooth-walled, subcylindrical, straight to curved, phialidic with visible periclinal thickening at the apex, 14–30 × 2–3 µm. Conidia hyaline, smooth, aseptate, straight, cylindrical with one end acute, granular and guttulate, (12.7–) 14.1–14.5 (–17.2) × (3.9–) 4.5–4.7 (–5.5) µm. Appressoria single, medium to dark brown, ovoid with an undulate margin, (7.2–) 8.1–8.3 (–9.5) × (5.4–) 6.5–6.7 (–7.6) µm.

Mycelia on mandarin rind were colourless to white. Conidiomata salmon, smooth. Conidia hyaline, smooth-walled, aseptate, straight, cylindrical with one end acute, granular and guttulate, (12.9–) 14.7–15.1 (–16.1) × (4.3–) 4.8–5 (–5.4) µm.

Culture characteristics: Colonies on SNA flat, entire margin, hyaline, 45–55 mm diam in 7 d. Colonies on PDA 65–75 mm in 7 d; pale yellow to white aerial mycelia, changing to grey in the centre, reverse have a uniform concentric ring with pinkish outside and inside pale grey to grey in the centre. Colonies on MEA flat, entire margin, white aerial mycelia, 52–78 mm in 7 d.

Notes: ***Colletotrichum australianum*** is phylogenetically close to *C. queenslandicum* but are separable using *ApMat* and *gs* sequences. The closest match in a Blastn search with the *gs* sequence was GenBank KP703693, *C. queenslandicum* strain CPC 17123, with 98 % identity.

### 3.4. Pathogenicity Assay

For the fruit bioassay, *C. australianum, C. fructicola, C. theobromicola, Colletotrichum* sp., and *C. karstii* developed brown lesions on wounded orange fruits. *Colletotrichum karstii* had the highest infection incidence at 100%, while the *C. gloeosporioides* isolate did not cause obvious symptoms (Table 4). None of the *Colletotrichum* species were able to infect non-wounded orange fruit.

For the leaf bioassay, *C. karstii* developed lesions on both orange and lemon leaves, while *C. theobromicola* only developed lesions on lemon leaves (Table 4). Other *Colletotrichum* isolates did not cause obvious symptoms on both orange and lemon leaves.

In the petal bioassay, all isolates infected orange petals.

## 4. Discussion

Six *Colletotrichum* species were identified from citrus stems, leaves, and fruits with anthracnose symptoms in Australia. *Colletotrichum australianum* was isolated from orange and mandarin fruit in Victoria, Australia, and identified and described as a new species causing anthracnose of citrus in Australia. Isolates from chili (*Capsicum annuum*) from Queensland and previously identified as *C. queenslandicum* [39] were also reidentified as *C. australianum*. Phylogenetic analyses clearly showed *C. australianum* to be a new species closely related to *C. queenslandicum*. There were also differences in morphological characters between these two species. The *ApMat* and *gs* sequences clearly distinguished *C. australianum*. These genes are considered as informative markers to identify species within the *C. gloeosporioides* species complex [10,36,51,53].

*Colletotrichum gloeosporioides sensu lato* was the most frequently isolated in diseased citrus. There was no preference for a particular *Citrus* sp. or infected organ tissue. *Colletotrichum gloeosporioides* was isolated from various citrus species, including cumquat, finger lime, grapefruit, lemon, lime, mandarin, orange, Persian lime, and Tahitian lime. *Colletotrichum gloeosporioides* was previously cultured from lemon (*Citrus limon*) and orange (*Citrus sinensis*) in Australia [37]. The isolate VPRI 10347 from lemon from Victoria and previously identified as *C. nymphaeae* [37] was also reidentified as *C. gloeosporioides*. The prevalence of *Colletotrichum* species that cause anthracnose of citrus in Australia, is in accordance with recent global studies on the major cause of anthracnose of citrus [8,11,12,13,14,19,20,21,23,24,27].

This is the first report in Australia of *Colletotrichum siamense* being associated with citrus anthracnose. *Colletotrichum siamense* was isolated from lemon fruit and finger lime fruit and has been recorded as a pathogen of a broad range of plants in Australia [37,39]. *Colletotrichum siamense* was previously reported to be isolated from catmon (*Citrus pennivesiculata*) in Bangladesh and Egypt, mandarin (*C. reticulata* Blanco cv. Shiyue Ju) in China, and mandarin (*C. reticulata* cv. Kinnow) in Pakistan [11,31,32,54]. *Colletotrichum siamense* isolate BRIP 54270b was collected in 2011 in Queensland, suggesting *C. siamense* has been a citrus pathogen for at least 10 years in Australia. However, both *C. siamense* isolates were collected from citrus fruits, and no *C. siamense* isolate was found on citrus leaves or stems, suggesting *C. siamense* is more likely to be a postharvest pathogen of citrus in Australia.

*Colletotrichum theobromicola* is for the first time reported as a pathogen of citrus. *Colletotrichum theobromicola* was isolated from lime fruit from Queensland but was recently neotypified from cacao tree (*Theobroma cacao*) in Panama [36]. *Colletotrichum theobromicola* has been recorded as a pathogen of a broad range of plants in Australia including jointvetch (*Aeschynomene falcata*), arabica coffee (*Coffea arabica*), olive (*Olea europaea*), pomegranate (*Punica granatum*), stylo (*Stylosanthes guianensis*), and sticky stylo (*Stylosanthes viscosa*) [37].

*Colletotrichum fructicola* was reported for the first time, associated with anthracnose symptoms from mandarin fruit in Australia. Isolate BRIP 65028 from Tahitian lime growing in Queensland was previously identified as *C. fructicola* in 2018 [38]. *Colletotrichum fructicola* was also isolated from avocado (*Persea americana*) in Australia [37]. In China, *C. fructicola* was reported to be associated with bergamot orange (*Citrus bergamia*), pomelo (*C. grandis*), mandarin (*C. reticulata* cv. nanfengmiju), oranges (*C. sinensis*), and kumquat (*Fortunella margarita*) [26,27,30]. *Colletotrichum* *fructicola* was found to cause both preharvest and postharvest citrus disease in Australia.

*Colletotrichum karstii* was the second dominant pathogen and was isolated from infected orange and lemon leaves in both New South Wales and Victoria. *Colletotrichum karstii* is the only species in the boninense species complex found to be associated with citrus anthracnose in Australia. Three *C. karstii* isolates were collected from orange leaves in the 1970s and were maintained in State fungaria, suggesting *C. karstii* has been a citrus pathogen for over 50 years in Australia but was misidentified as *C. gloeosporioides*. *Colletotrichum karstii* was reported to infect citrus and to have a wide global distribution [8,11,13,16,23,25,26,27,28,29]. Previously, *C. karstii* was reported from other hosts such as black plum (*Diospyros australis*), strawberry (*Fragaria* x *ananassa*), and banana (*Musa banksia*) in Australia [37].

Six *Colletotrichum* isolates from chili (*Capsicum annuum*) that had been previously identified as causing anthracnose fruit rot of chili in Brisbane, Queensland, Australia [39], were also identified as *C. australianum.* These six *Colletotrichum* isolates were morphologically similar to *C. australianum* from citrus rather than the type specimen of *C. queenslandicum* (ICMP 1778), which was originally isolated from infected papaya. The identification of *C. australianum* from diverse hosts such as orange, mandarin, and chili, suggests that *C. australianum* may have a broad host range. Further studies are required on the host range of this pathogen, which may have biosecurity implication for the export of Australian fruit. The occurrence of *C. australianum* in both Victoria and Queensland indicates the wide geographic spread across different climatic zones in Australia.

The species identification of *Colletotrichum* isolates based on *ApMat* and *gs* gene sequences were as similar as the results from phylogenetic analysis of seven-gene combination, proving that the locus *ApMat* was effective in identifying *Colletotrichum* species within the gloeosporioides species complex. The phylogenetic analysis of combined *ApMat* and *gs* sequences can identify species within the gloeosporioides species complex [10,47,51,53]. The efficiency of the *ApMat* gene to identify species was also supported by Sharma et al. [55] and Sharma, Pinnaka, and Shenoy [56], who differentiated *Colletotrichum* isolates in India. The isolate VPRI 10347 was identified to be *Colletotrichum nymphaeae* in Shivas et al. [37] based on single *tub2* sequence. However, in this study, *ApMat* and *gs* gene sequences identified isolate VPRI 10347 as *C. gloeosporioides*, same as the result from phylogenetic analysis of the seven-gene combination. However, the limitation of using the *ApMat* gene in constructing phylogenetic trees is that several reference *Colletotrichum* species in the gloeosporioides species complex in GenBank have not been sequenced for *ApMat*. For example, the isolate VPRI 43083 was phylogenetically close to *C. grevilleae* and *C. grossum* based on analysis of combined ITS and *tub2* gene sequences (Supplementary Figure S1) but due to a lack of *ApMat* sequence of *C. grevilleae* and *C. grossum,* these species were not included in either the seven-gene nor the two-gene trees, whereas VPRI 43083 was identified as *C. theobromicola* based on seven gene combination and two gene combination analyses with high bootstrap value. Due to a lack of replicate isolates, as well as a lack of reference sequences, especially *ApMat* gene data of *Colletotrichum* species close to BRIP 58074a, the unknown *Colletotrichum* sp. (BRIP 58074a) isolate cannot be further described taxonomically or phylogenetically at this stage.

*Colletotrichum acutatum* has been reported from lemon (DAR 80516, from Tasmania in 2009, and DAR 72160, from NSW in 1998) previously [38]. However, *C. acutatum* was not found in this study. The two *C. acutatum* isolates were identified based on morphology but have not been confirmed by molecular analysis. Gene sequences of isolates DAR 80516 and DAR 72160 should be analysed to accurately identify these two isolates.

Pathogenicity tests of five *Colletotrichum* species from citrus showed that all species except for *C. gloeosporioides* were capable of infecting wounded fruit. In contrast, none of the five *Colletotrichum* species caused disease on the non-wounded fruit. These results are consistent with previous reports where wound inoculated citrus fruits were used in postharvest pathogenicity testing of *Colletotrichum* species [8,27]. Variable maturity of the fruit may also be a reason for lack of infection. Mature fruits are reported to be more sensitive to *Colletotrichum* species [57]. The fruit used for inoculation in this study may not have been fully mature, although they were selected based on the colour of mature fruit; thus, they were not conducive for *Colletotrichum* spores to attach to the cuticle, germinate, and form appressoria prior to infection.

Different *Colletotrichum* species had various degrees of aggressiveness on wounded orange fruit and non-wounded orange and lemon leaves. *Colletotrichum karstii* was the most aggressive species when infecting orange fruit and orange and lemon leaves. The variable aggressiveness of different *Colletotrichum* species has been reported by Guarnaccia et al. [8]. *Colletotrichum gloeosporioides* isolate VPRI 43076 was non-pathogenic on fruit and leaves but was pathogenic on orange petals. Conversely, Guarnaccia et al. [8] reported *C. gloeosporioides* to be the most aggressive species when infecting orange fruit. Pathogenic variation has been reported within populations of a *Colletotrichum* species [10,58,59]. Hence, VPRI 43076 was likely to have been an isolate of *Colletotrichum gloeosporioides*, which had weak aggressiveness on citrus fruit. Further assessment of pathogenicity of isolates from each species needs to be undertaken to determine the variability of aggressiveness.

## 5. Conclusions

Six *Colletotrichum* spp. were identified to cause anthracnose of citrus in Australia that included one novel species *C. australianum*, and one undetermined species. In addition, this was the first report of *C. theobromicola* as a pathogen of citrus globally, and the first report of *C. karstii* and *C.* *siamense* to be associated with citrus anthracnose in Australia.

## Figures and Tables

**Table 1:** Strains of *Colletotrichum* species used in the phylogenetic analyses with details of host and location, and GenBank accession numbers of the sequences.

Species	Accession Number	Host	Location	GenBank Accession Numbers							
				ITS	*GAPDH*	*ACT*	*TUB2*	*gs*	*ApMat*	*CHS-1*	*HIS3*
Gloeosporioides complex											
*C. aenigma*	ICMP 18608 *	*Persea americana*	Israel	JX010244	JX010044	JX009443	JX010389	JX010078	KM360143	JX009774	–
*C. aeschynomenes*	ICMP 17673; ATCC 201874 *	*Aeschynomene virginica*	USA	JX010176	JX009930	JX009483	JX010392	JX010081	KM360145	JX009799	–
*C. alatae*	ICMP 17919 *	*Dioscorea alata*	India	JX010190	JX009990	JX009471	JX010383	JX010065	KC888932	JX009837	–
*C. alienum*	ICMP 12071 *	*Malus domestica*	New Zealand	JX010251	JX010028	JX009572	JX010411	JX010101	KM360144	JX009882	–
*C. asianum*	ICMP 18580; CBS 130418 *	*Coffea arabica*	Thailand	FJ972612	JX010053	JX009584	JX010406	JX010096	FR718814	JX009867	–
*C. aotearoa*	ICMP 18537 *	*Coprosma* sp.	New Zealand	JX010205	JX010005	JX009564	JX010420	JX010113	KC888930	JX009853	–
*C. artocarpicola*	MFLUCC 18–1167 *	*Artocarpus heterophyllus*	Thailand	MN415991	MN435568	MN435570	MN435567	–	–	MN435569	–
*C. australianum*	VPRI 43074; UMC001	*Citrus reticulata*	Australia, Vic	MG572137	MG572126	MK473452	MG572148	MG572159	MG572170	MW091986	–
	VPRI 43075; UMC002 *	*Citrus sinensis*	Australia, Vic	MG572138	MG572127	MN442109	MG572149	MG572160	MG572171	MW091987	–
	BRIP 63695	*Capsicum annuum*	Australia	KU923677	MN442115	MN442105	KU923693	KU923737	KU923727	MW092000	–
	BRIP 63696	*Capsicum annuum*	Australia	KU923678	–	–	KU923694	KU923738	KU923728	–	–
	BRIP 63697	*Capsicum annuum*	Australia	KU923679	–	–	KU923695	KU923739	KU923729	–	–
	BRIP 63698	*Capsicum annuum*	Australia	KU923680	MN442116	MN442106	KU923696	KU923740	KU923730	MW092001	–
	BRIP 63699	*Capsicum annuum*	Australia	KU923681	MN442117	MN442107	KU923697	KU923741	KU923731	MW092002	–
	BRIP 63700	*Capsicum annuum*	Australia	KU923682	MN442118	MN442108	KU923698	KU923742	KU923732	MW092003	–
*C. camelliae*	CGMCC 3.14925 *	*Camellia sinensis*	China	KJ955081	KJ954782	KJ954363	KJ955230	KJ954932	KJ954497	–	–
*Glomella cingulate* f. sp. *camelliae*	ICMP 10643 *	*Camellia × williamsii*	UK	JX010224	JX009908	JX009540	JX010436	JX010119	KJ954625	JX009891	–
*C. changpingense*	MFLUCC 15-0022	*Fragaria ×ananassa*	China	KP683152	KP852469	KP683093	KP852490	–	–	KP852449	–
*C. chrysophilum*	CMM4268 *	*Musa* sp.	Brazil	KX094252	KX094183	KX093982	KX094285	KX094204	–	KX094083	–
*C. conoides*	CAUG17 *	*Capsicum annuum*	China	KP890168	KP890162	KP890144	KP890174	–	–	KP890156	–
*C. cordylinicola*	MFLUCC 090551; ICMP 18579 *	*Cordyline fruticosa*	Thailand	JX010226	JX009975	HM470235	JX010440	JX010122	JQ899274	JX009864	–
*C. clidemiae*	ICMP 18658 *	*Clidemia hirta*	USA, Hawaii	JX010265	JX009989	JX009537	JX010438	JX010129	KC888929	JX009877	–
*C. endophytica*	CAUG28	*Capsicum annuum*	China	KP145441	KP145413	KP145329	KP145469	–	–	KP145385	–
*C. fructicola*	ICMP 18581; CBS 130416 *	*Coffea arabica*	Thailand	JX010165	JX010033	FJ907426	JX010405	JX010095	JQ807838	JX009866	–
	LC2923; LF130	*Camellia sinensis*	China	KJ955083	KJ954784	KJ954365	KJ955232	KJ954934	KJ954499	–	–
	VPRI 43079; UMC006	*Citrus reticulata*	Australia, Qld	MG572142	MG572131	MK473454	MG572153	MG572164	MG572175	MW091991	–
	BRIP 65028a; VPRI 43034; B03-43034	*Citrus latifolia*	Australia, Qld	MK470007	MK470025	MK470097	MK470061	MK470043	MK470079	MW091983	–
*C. fructicola* (syn. *C. ignotum*)	ICMP 18646	*Tetragastris panamensis*	Panama	JX010173	JX010032	JX009581	JX010409	JX010099	JQ807839	JX009874	–
*C. fructivorum*	CBS 133125 *	*Vaccinium macrocarpon*	USA	JX145145	–	–	JX145196	–	–	–	–
*C. gloeosporioides*	IMI 356878; ICMP 17821; CBS 112999 *	*Citrus sinensis*	Italy	JX010152	JX010056	JX009531	JX010445	JX010085	JQ807843	JX009818	–
	LC3110; LF318	*Camellia sinensis*	China	KJ955127	KJ954828	KJ954407	KJ955275	KJ954978	KJ954541	–	–
	LC3312; LF534	*Camellia sinensis*	China	KJ955158	KJ954859	KJ954434	KJ955305	KJ955009	KJ954569	–	–
	LC3382; LF604	*Camellia sinensis*	China	KJ955176	KJ954877	KJ954450	KJ955323	KJ955026	KJ954584	–	–
	LC3686; LF916	*Camellia sinensis*	China	KJ955226	KJ954927	KJ954493	KJ955371	KJ955076	KJ954629	–	–
	VPRI 43076; UMC003	*Citrus sinensis*	Australia, Vic	MG572139	MG572128	MN442110	MG572150	MG572161	MG572172	MW091988	–
	VPRI 43078; UMC005	*Citrus aurantifolia*	Australia, Qld	MG572141	MG572130	MN442111	MG572152	MG572163	MG572174	MW091990	–
	VPRI 43080; UMC007	*Citrus reticulata*	Australia, Qld	MG572143	MG572132	MK473455	MG572154	MG572165	MG572176	MW091992	–
	VPRI 43081; UMC008	*Citrus reticulata*	Australia, Qld	MG572144	MG572133	MN442112	MG572155	MG572166	MG572177	MW091993	–
	VPRI 43082; UMC009	*Citrus reticulata*	Australia, Qld	MG572145	MG572134	MN442113	MG572156	MG572167	MG572178	MW091994	–
	VPRI 43084; UMC011	*Citrus japonica*	Australia, Vic	MG572147	MG572136	MN442114	MG572158	MG572169	MG572180	MW091996	–
	VPRI 43648; UMC012	*Citrus sinensis*	Australia, Vic	MW081160	MW081163	MW081166	MW081169	MW081175	MW081172	MW091997	–
	VPRI 43649; UMC013	*Citrus limon*	Australia, Vic	MW081161	MW081164	MW081167	MW081170	MW081176	MW081173	MW091998	–
	VPRI 43650; UMC014	*Citrus japonica*	Australia, Vic	MW081162	MW081165	MW081168	MW081171	MW081177	MW081174	MW091999	–
	VPRI 10312; A01-10312	*Citrus sinensis*	Australia, Vic	MK469996	MK470014	MK470086	MK470050	MK470032	MK470068	MW091972	–
	VPRI 10347; A02-10347; BRIP 54771	*Citrus limon*	Australia, Vic	MK469997	MK470015	MK470087	MK470051; KU221374	MK470033	MK470069	MW091973	–
	WAC 12803; BRIP 63680a; VPRI 43024; A05-43024	*Citrus sinensis*	Australia, WA	MK469998	MK470016	MK470088	MK470052	MK470034	MK470070	MW091974	–
	BRIP 66210a; VPRI 43026; A07-43026	*Citrus reticulata*	Australia, SA	MK470000	MK470018	MK470090	MK470054	MK470036	MK470072	MW091976	–
	BRIP 66210b; VPRI 43027; A08-43027	*Citrus reticulata*	Australia, SA	MK470001	MK470019	MK470091	MK470055	MK470037	MK470073	MW091977	–
	BRIP 28546a; VPRI 43028; A09-43028	*Citrus sinensis* Navel	Australia, Qld	MK470002	MK470020	MK470092	MK470056	MK470038	MK470074	MW091978	–
	BRIP 28754a; VPRI 43030; A11-43030	*Citrus reticulata*	Australia, Qld	MK470003	MK470021	MK470093	MK470057	MK470039	MK470075	MW091979	–
	BRIP 53157d; VPRI 43031; A12-43031	*Citrus aurantifolia* Tahiti	Australia, Qld	MK470004	MK470022	MK470094	MK470058	MK470040	MK470076	MW091980	–
	BRIP 66135a; VPRI 43032; B01-43032	*Citrus reticulata* Imperial Blanco	Australia, Qld	MK470005	MK470023	MK470095	MK470059	MK470041	MK470077	MW091981	–
	BRIP 28831a; VPRI 43033; B02-43033	*Citrus sinensis*	Australia, Qld	MK470006	MK470024	MK470096	MK470060	MK470042	MK470078	MW091982	–
	VPRI 42955; G01-42955	*Citrus limon*	Australia, NSW	MK470008	MK470026	MK470098	MK470062	MK470044	MK470080	MW091984	–
	VPRI 42956; H01-42956	*Citrus sinensis*	Australia, NSW	MK470009	MK470027	MK470099	MK470063	MK470045	MK470081	MW091985	–
*C. grevilleae*	CBS 132879 *	*Grevillea* sp.	Italy	KC297078	KC297010	KC296941	KC297102	KC297033	–	KC296987	–
*C. grossum*	CGMCC3.17614T; CAUG7 *	*Capsicum* sp.	China	KP890165	KP890159	KP890141	KP890171	–	–	KP890153	–
	CAU31	*Capsicum* sp.	China	KP890166	KP890160	KP890142	KP890172	–	–	KP890154	–
	CAUG32	*Capsicum* sp.	China	KP890167	KP890161	KP890143	KP890173	–	–	KP890155	–
*C. hebeiense*	MFLUCC13-0726 *	*Vitis vinifera* cv. *Cabernet Sauvignon*	China	KF156863	KF377495	KF377532	KF288975	–	–	KF289008	–
*C. helleniense*	CPC 26844; CBS 142418 *	*Poncirus trifoliata*	Greece	KY856446	KY856270	KY856019	KY856528	–	–	KY856186	–
*C. henanense*	LC3030; CGMCC 3.17354; LF238 *	*Camellia sinensis*	China	KJ955109	KJ954810	KM023257	KJ955257	KJ954960	KJ954524	–	–
*C. horii*	ICMP 10492 *	*Diospyros kaki*	Japan	GQ329690	GQ329681	JX009438	JX010450	JX010137	JQ807840	JX009752	–
*C. hystricis*	CPC 28153; CBS 142411 *	*Citrus hystrix*	Italy	KY856450	KY856274	KY856023	KY856532	–	–	KY856190	–
*C. jiangxiense*	LF687 *	*Camellia sinensis*	China	KJ955201	KJ954902	KJ954471	KJ955348	KJ955051	KJ954607	–	–
*C. cigarro*	ICMP 18534	*Kunzea ericoides*	New Zealand	JX010227	JX009904	JX009473	JX010427	JX010116	HE655657	JX009765	–
*C. kahawae*	IMI 319418; ICMP 17816 *	*Coffea arabica*	Kenya	JX010231	JX010012	JX009452	JX010444	JX010130	JQ894579	JX009813	–
*C. musae*	ICMP 19119; CBS 116870 *	*Musa* sp.	USA	JX010146	JX010050	JX009433	HQ596280	JX010103	KC888926	JX009896	–
	ICMP 17817	*Musa sapientum*	Kenya	JX010142	JX010015	JX009432	JX010395	JX010084	–	JX009815	–
*C. nupharicola*	ICMP 18187 *	*Nuphar lutea* subsp. *polysepala*	USA	JX010187	JX009972	JX009437	JX010398	JX010088	JX145319	JX009835	–
*C. pandanicola*	MFLUCC 17-0571	*Pandanaceae*	Thailand	MG646967	MG646934	MG646938	MG646926	–	–	MG646931	–
*C. proteae*	CBS 132882 *	*Protea* sp.	South Africa	KC297079	KC297009	KC296940	KC297101	KC297032	–	KC296986	–
*C. psidii*	ICMP 19120 *	*Psidium* sp.	Italy	JX010219	JX009967	JX009515	JX010443	JX010133	KC888931	JX009901	–
*C. queenslandicum*	ICMP 1778 *	*Carica papaya*	Australia	JX010276	JX009934	JX009447	JX010414	JX010104	KC888928	JX009899	–
	CPC 17123	*Syzygium australa*	Australia	KP703357	KP703282	–	KP703439	KP703693	KP703778	–	–
	ICMP 18705	*Coffea* sp.	Fiji	JX010185	JX010036	JX009490	JX010412	JX010102	–	JX009890	–
	CMM3233	*Anacardium occidentale*	Brazil, Pernambuco state	–	MF110849	–	MF111058	MF110996	MF110639	–	–
	CMM3241	*Anacardium occidentale*	Brazil, Pernambuco state	–	MF110848	–	MF111059	MF111000	MF110642	–	–
	CMM3236	*Anacardium occidentale*	Brazil, Pernambuco state	–	MF110850	–	MF111060	MF110997	MF110640	–	–
	CMM3240	*Anacardium occidentale*	Brazil, Pernambuco state	–	MF110852	–	MF111061	MF110999	MF110644	–	–
	CMM3237	*Anacardium occidentale*	Brazil, Pernambuco state	–	MF110853	–	MF111062	MF110998	MF110641	–	–
	CMM3242	*Anacardium occidentale*	Brazil, Pernambuco state	–	–	–	MF111063	MF111001	MF110643	–	–
*C. rhexiae*	CBS 133134 *	*Rhexia virginica*	USA	JX145128	–	–	JX145179	–	–	–	–
*C. salsolae*	ICMP 19051 *	*Salsola tragus*	Hungary	JX010242	JX009916	JX009562	JX010403	JX010093	KC888925	JX009863	–
*C. siamense*	ICMP 18578 CBS 130417 *	*Citrus arabica*	Thailand	JX010171	JX009924	FJ907423	JX010404	JX010094	JQ899289	JX009865	–
	VPRI 43077; UMC004	*Citrus limon*	Australia, NSW	MG572140	MG572129	MK473453	MG572151	MG572162	MG572173	MW091989	–
	BRIP 54270b; VPRI 43029; A10-43029	*Citrus australasica*	Australia, Qld	MK469995	MK470013	MK470085	MK470049	MK470031	MK470067	MW091971	–
*C. siamense* (syn. *C. jasmini-sambac*)	CBS 130420; ICMP 19118	*Jasminum sambac*	Vietnam	HM131511	HM131497	HM131507	JX010415	JX010105	JQ807841	JX009895	–
*C. siamense* (syn. *C. hymenocallidis*)	CBS 125378; ICMP 18642; LC0043	*Hymenocallis americana*	China	JX010278	JX010019	GQ856775	JX010410	JX010100	JQ899283	GQ856730	–
*C. siamense* (syn. *C. murrayae*)	GZAAS 5.09506	*Murraya* sp.	China	JQ247633	JQ247609	JQ247657	JQ247644	JQ247621	–	–	–
*C. syzygicola*	DNCL021; MFLUCC 10-0624 *	*Syzygium samarangense*	Thailand	KF242094	KF242156	KF157801	KF254880	KF242125	–	–	–
*C. temperatum*	CBS 133122 *	*Vaccinium macrocarpon*	USA	JX145159	–	–	JX145211	–	–	–	–
*C. theobromicola*	ICMP 18649; CBS 124945 *	*Theobroma cacao*	Panama	JX010294	JX010006	JX009444	JX010447	JX010139	KC790726	JX009869	–
*C. theobromicola* (syn. *C. fragariae*)	ICMP 17927; CBS 142.31; MTCC 10325T	*Fragaria × ananassa*	USA	JX010286	JX010024	JX009516	JX010373	JX010064	JQ807844	JX009830	–
	VPRI 43083; UMC010	*Citrus aurantifolia*	Australia, Qld	MG572146	MG572135	MK473456	MG572157	MG572168	MG572179	MW091995	–
*C. ti*	ICMP 4832 *	*Cordyline* sp.	New Zealand	JX010269	JX009952	JX009520	JX010442	JX010123	KM360146	JX009898	–
*C. tropicale*	ICMP 18653; CBS 124949 *	*Theobroma cacao*	Panama	JX010264	JX010007	JX009489	JX010407	JX010097	KC790728	JX009870	–
*C. viniferum*	GZAAS 5.08601 *	*Vitis vinifera,* cv. *‘Shuijing’*	China	JN412804	JN412798	JN412795	JN412813	JN412787	–	–	–
*C. wuxiense*	CGMCC 3.17894 *	*Camellia sinensis*	China	KU251591	KU252045	KU251672	KU252200	KU252101	KU251722	KU251939	–
*C. xanthorrhoeae*	BRIP 45094; ICMP 17903; CBS 127831 *	*Xanthorrhoea preissii*	Australia	JX010261	JX009927	JX009478	JX010448	JX010138	KC790689	JX009823	–
*Colletotrichum* sp.	BRIP 58074a; VPRI 43025; A06-43025	*Citrus australasica*	Australia, Qld	MK469999	MK470017	MK470089	MK470053	MK470035	MK470071	MW091975	–
Boninense complex											
*C. annellatum*	CBS 129826 *	*Hevea brasiliensis*	Colombia	JQ005222	–	JQ005570	JQ005656	–	–	JQ005396	JQ005483
*C. beeveri*	CBS 128527 *	*Brachyglottis repanda*	New Zealand	JQ005171	–	JQ005519	JQ005605	–	–	JQ005345	JQ005432
*C. boninense*	ICMP 17904; CBS 123755 *	*Crinum asiaticum* ‘Sinicum’	Japan	JQ005153	–	JQ005501	JQ005588	–	–	JQ005327	JQ005414
*C. brassicicola*	CBS 101059	*Brassica oleracea* var. *gemmifera*	New Zealand	JQ005172	–	JQ005520	JQ005606	–	–	JQ005346	JQ005433
*C. brasiliense*	CBS 128501*	*Passiflora edulis*	Brazil	JQ005235	–	JQ005583	JQ005669	–	–	JQ005409	JQ005496
*C. catinaense*	CBS 142417; CPC 27978 *	*Citrus reticulata*	Italy, Catania	KY856400	–	KY855971	KY856482	–	–	KY856136	KY856307
*C. citricola*	CBS 134228 *	*Citrus unchiu*	China	KC293576	–	KC293616	KC293656	–	–	KY856140	KY856311
*C. constrictum*	CBS 128504	*Citrus limon*	New Zealand	JQ005238	–	JQ005586	JQ005672	–	–	JQ005412	KY856313
*C. colombiense*	CBS 129818 *	*Passiflora edulis*	Colombia	JQ005174	–	JQ005522	JQ005608	–	–	JQ005348	JQ005435
*C. cymbidiicola*	IMI 347923 *	*Cymbidium* sp.	Australia	JQ005166	–	JQ005514	JQ005600	–	–	JQ005340	JQ005427
*C. dacrycarpi*	CBS 130241 *	*Dacrycarpus dacrydioides*	New Zealand	JQ005236	–	JQ005584	JQ005670	–	–	JQ005410	JQ005497
*C. hippeastri*	CBS 125376 *	*Hippeastrum vittatum*	China	JQ005231	–	JQ005579	JQ005665	–	–	JQ005405	JQ005492
*C. karstii*	CBS 126532	*Citrus* sp.	South Africa	JQ005209	–	JQ005557	JQ005643	–	–	JQ005383	JQ005470
	CBS 128551	*Citrus* sp.	New Zealand	JQ005208	–	JQ005556	JQ005642	–	–	JQ005382	JQ005469
	CBS 129829	*Gossypium hirsutum*	Germany	JQ005189	–	JQ005537	JQ005623	–	–	JQ005363	JQ005450
	CPC 27853	*Citrus sinensis*	Italy, Catania	KY856461	–	KY856034	KY856543	–	–	KY856202	KY856377
	CPC 31139	*Citrus sinensis*	Italy, Catania	KY856467	–	KY856040	KY856549	–	–	KY856208	KY856383
	CBS 129833	*Musa* sp.	Mexico	JQ005175	–	JQ005523	JQ005609	–	–	JQ005349	JQ005436
	CBS 861.72	*Bombax aquaticum*	Brazil	JQ005184	–	JQ005532	JQ005618	–	–	JQ005358	JQ005445
	DAR 25017a; VPRI 42941; D02-42941	*Citrus sinensis* Valencia	Australia, NSW	MK470103	–	MK470109	MK470106	–	–	MK470115	MK470112
	DAR 29821a; VPRI 42943; F02-42943	*Citrus sinensis* Valencia	Australia, NSW	MK470104	–	MK470110	MK470107	–	–	MK470116	MK470113
	DAR 29826a; VPRI 42944; G02-42944	*Citrus sinensis* Valencia	Australia, NSW	MK470105	–	MK470111	MK470108	–	–	MK470117	MK470114
	VPRI 43651; UMC015	*Citrus limon*	Australia, Vic	MW081178	–	MW081186	MW081182	–	–	MW081190	MW081194
	VPRI 43652; UMC016	*Citrus sinensis*	Australia, Vic	MW081179	–	MW081187	MW081183	–	–	MW081191	MW081195
	VPRI 43653; UMC017	*Citrus sinensis*	Australia, Vic	MW081180	–	MW081188	MW081184	–	–	MW081192	MW081196
	VPRI 43654; UMC018	*Citrus sinensis*	Australia, Vic	MW081181	–	MW081189	MW081185	–	–	MW081193	MW081197
*C. limonicola*	CBS 142410; CPC 31141 *	*Citrus limon*	Malta, Gozo	KY856472	–	KY856045	KY856554	–	–	KY856213	KY856388
*C. novae-zelandiae*	CBS 128505 *	*Capsicum annuum*	New Zealand	JQ005228	–	JQ005576	JQ005662	–	–	JQ005402	JQ005489
*C. oncidii*	CBS 129828 *	*Oncidium* sp.	Germany	JQ005169	–	JQ005517	JQ005603	–	–	JQ005343	JQ005430
*C. parsonsiae*	CBS 128525 *	*Parsonsia capsularis*	New Zealand	JQ005233	–	JQ005581	JQ005667	–	–	JQ005407	JQ005494
*C. petchii*	CBS 378.94 *	*Dracaena marginata*	Italy	JQ005223	–	JQ005571	JQ005657	–	–	JQ005397	JQ005484
*C. phyllanthi*	CBS 175.67 *	*Phyllanthus acidus*	India	JQ005221	–	JQ005569	JQ005655	–	–	JQ005395	JQ005482
*C. torulosum*	CBS 128544 *	*Solanum melongena*	New Zealand	JQ005164	–	JQ005512	JQ005598	–	–	JQ005338	JQ005425
Truncatum complex											
*C. truncatum*	CBS 151.35 *	*Phaseolus lunatus*	USA	GU227862	–	GU227960	GU228156	–	–	GU228352	GU228058

Vic: Victoria, NSW: New South Wales, Qld: Queensland, WA: Western Australia, SA: South Australia. * Ex-holotype or ex-epitype cultures.

**Table 2:** Best-fit model for each gene locus selected by MrModeltest.

Dataset	Substitution Models						
	ITS	*tub2*	*act*	*chs-1*	*his3*		
**boninense complex**	SYM + I+G	HKY + I	HKY + G	GTR + G	HKY + I		
	ITS	*gapdh*	*tub2*	*act*	*ApMat*	*gs*	*chs-1*
**gloeosporioides complex**	SYM + I	HKY + I	SYM + I	HKY + I	HKY + G	GTR + G	K80 + G

**Table 3:** Morphological characters of *Colletotrichum* species.

Taxon	Conidial Length (μm)	Conidial Width (μm)	Conidial Shape	Growth Rate (mm/day) ^1^
*C. gloeosporioides*	(10.2–) 13.8–14.3 (–16.1)	(4.2–) 5.3–5.5 (–7.3)	Subcylindrical	10.4–10.8
*C. siamense*	(12.0–) 13.1–13.4 (–15.8)	(4.8–) 5.4–5.5 (–5.9)	Fusoid	10.9–11.5
*C. fructicola*	(12.7–) 14.2–14.6 (–17.1)	(4.6–) 5.1–5.2 (–5.7)	Cylindrical	10.5–11.1
*C. theobromicola*	(10.8–) 15.2–16 (–21.2)	(4.0–) 4.8–5 (–5.8)	Cylindrical	10.5–10.7
*Colletotrichum* sp.	(13.1–) 15.6–16 (–18.0)	(4.6–) 6.1–6.3 (–7.7)	Cylindrical	8.9–9.7
*C. karstii*	(11.3–) 13.2–13.6 (–14.8)	(6.4–) 7.1–7.3 (–8.3)	Cylindrical	9.4–9.6
New species	(12.7–) 14.1–14.5 (–17.2)	(3.9–) 4.5–4.7 (–5.5)	Cylindrical with one end acute	9.7–10.3
*C. queenslandicum* ^2^	(12–) 14.5–16.5 (–21.5)	(3.5–) 4.5–5 (–6)	Cylindric, straight, sometimes slightly constricted near center, ends broadly rounded	/

^1^ Seven *Colletotrichum* species incubated at 25 °C for 7 d. Colony growth was determined by measuring two diameters perpendicular to each other per plate and determining the average of six plates. ^2^ *C. queenslandicum* ICMP 1778, MycoBank MB563593 [36].

**Table 4:** Incidence of infection on Washington Navel orange fruit and leaves and Meyer lemon leaves by *Colletotrichum* species.

Culture	Fungus Species	Infection Incidence %			
		Fruit Bioassay (Wound)	Leaf Bioassay		Petal Bioassay
			Orange Leaf	Lemon Leaf	
VPRI 43075	*C. australianum* sp. nov.	95.8	0	0	100
VPRI 43076	*C. gloeosporioides*	0	0	0	100
VPRI 43079	*C. fructicola*	75	0	0	100
VPRI 43083	*C. theobromicola*	95.8	0	83.3	100
BRIP 58074a	*Colletotrichum* sp.	95.8	0	0	100
VPRI 43654	*C. karstii*	100	100	100	100

## Data Availability

Alignments generated during the current study are available in TreeBASE (accession http://purl.org/phylo/treebase/phylows/study/TB2:S27542). All sequence data are available in NCBI GenBank following the accession numbers in the manuscript.

## Supplementary Materials

The following are available online at https://www.mdpi.com/2309-608X/7/1/47/s1: Figure S1: Phylogram generated from maximum likelihood analysis of all available *Colletotrichum* species in the gloeosporioides species complex and the boninense species complex based on combined ITS and *tub2* sequences data, Table S1: Information of the 36 *Colletotrichum* isolates selected for phylogenetic analyses.
