# Supplementary figures and images for: Colletotrichum Species Causing Anthracnose of Citrus in Australia
Source: J Fungi (Basel). 2021 Jan 12;7(1):47. doi: 10.3390/jof7010047 (PMC7828153; doi:10.3390/jof7010047)

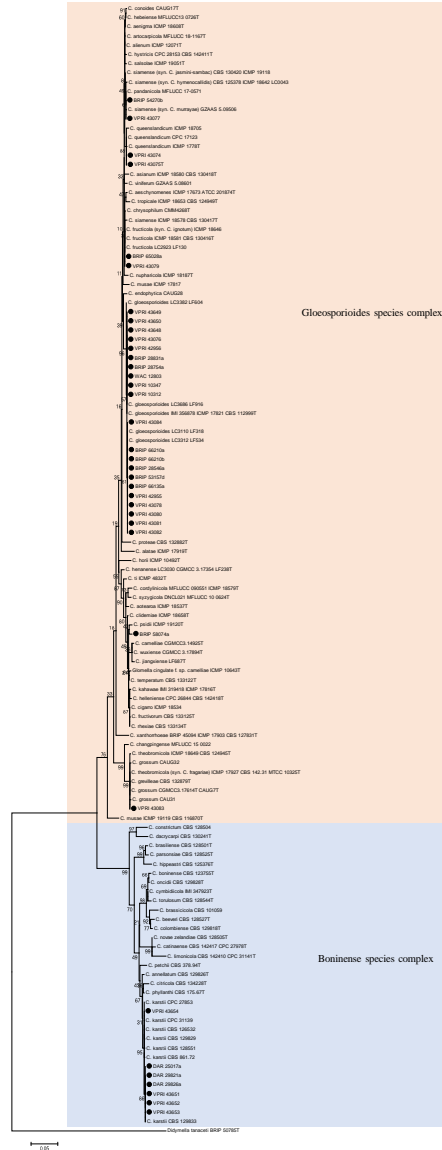

Supplement: Supplementary file 1 [file jof-07-00047-s001.zip › Figure S1.pdf]
